# Fecal Microbiome Transplantation from Children with Autism Spectrum Disorder Modulates Tryptophan and Serotonergic Synapse Metabolism and Induces Altered Behaviors in Germ-Free Mice

**DOI:** 10.1128/mSystems.01343-20

**Published:** 2021-04-06

**Authors:** Lu Xiao, Junyan Yan, Ting Yang, Jiang Zhu, Tingyu Li, Hong Wei, Jie Chen

**Affiliations:** a Children’s Nutrition Research Center, Children’s Hospital of Chongqing Medical University, Chongqing Key Laboratory of Childhood Nutrition and Health, Chongqing, China; b National Clinical Research Center for Child Health and Disorders, Ministry of Education Key Laboratory of Child Development and Disorders, Chongqing, China; c Laboratory Animal Department, College of Basic Medicine, Army Medical University, Chongqing, China; University of Hawaii at Manoa

**Keywords:** fecal microbiota transplantation (FMT), autism spectrum disorder (ASD), gut microbiota, tryptophan metabolism, serotonin metabolism

## Abstract

To determine the relationship of the gut microbiota and its metabolites with autism spectrum disorder (ASD)-like behaviors and preliminarily explore the potential molecular mechanisms, the fecal microbiota from donors with ASD and typically developing (TD) donors were transferred into germ-free (GF) mice to obtain ASD-FMT mice and TD-FMT mice, respectively. Behavioral tests were conducted on these mice after 3 weeks. 16S rRNA gene sequencing of the cecal contents and untargeted metabolomic analysis of the cecum, serum, and prefrontal cortex were performed. Untargeted metabolomics was also used to analyze fecal samples of TD and ASD children. Western blotting detected the protein expression levels of tryptophan hydroxylase 1 (TPH1), serotonin transporter (SERT), and serotonin 1A receptor (5-HT1AR) in the colon and TPH2, SERT, and 5-HT1AR in the prefrontal cortex of mice. ASD-FMT mice showed ASD-like behavior and a microbial community structure different from that of TD-FMT mice. Tryptophan and serotonin metabolisms were altered in both ASD and TD children and ASD-FMT and TD-FMT mice. Some microbiota may be related to tryptophan and serotonin metabolism. Compared with TD-FMT mice, ASD-FMT mice showed low SERT and 5-HT1AR and high TPH1 expression levels in the colon. In the prefrontal cortex, the expression levels of TPH2 and SERT were increased in the ASD-FMT group relative to the TD-FMT group. Therefore, the fecal microbiome of ASD children can lead to ASD-like behaviors, different microbial community structures, and altered tryptophan and serotonin metabolism in GF mice. These changes may be related to changes in some key proteins involved in the synthesis and transport of serotonin.

**IMPORTANCE** The relationship between the gut microbiota and ASD is not yet fully understood. Numerous studies have focused on the differences in intestinal microbial and metabolism profiles between TD and ASD children. However, it is still not clear if these microbes and metabolites cause the development of ASD symptoms. Here, we collected fecal samples from TD and ASD children, transplanted them into GF mice, and found that the fecal microbiome of ASD children can lead to ASD-like behaviors, different microbial community structures, and altered tryptophan and serotonin metabolism in GF mice. We also demonstrated that tryptophan and serotonin metabolism was also altered in ASD and TD children. Together, these findings confirm that the microbiome from children with ASD may lead to ASD-like behavior of GF mice through metabolites, especially tryptophan and serotonin metabolism.

## INTRODUCTION

Autism spectrum disorder (ASD) is a category of neurodevelopmental disorders that are characterized by social and communication impairments and restricted or repetitive behaviors (1). Although genetic and environmental factors have been associated with an increased risk of ASD (2), the exact etiology and pathophysiology remain unknown (3). Recent research has shown that the complex community of intestinal microorganisms and their metabolism may contribute to the pathogenesis of ASD via the microbiota-gut-brain axis (4), a bidirectional communication system that enables communication between the gut microbes and the brain (5, 6). Animal experiments have revealed that intestinal microbes are closely related to social behaviors, anxiety, memory, and cognition in mice (7). Furthermore, compared with specific-pathogen-free (SPF) mice, germ-free (GF) mice show social communication disorders and increased anxiety behaviors, and these abnormal social behaviors are corrected after the mice are colonized with normal microbiota (8, 9). Thus, disturbances of the gut microbiota may contribute to the onset of psychiatric disorders, including ASD.

The relationship between the gut microbiota and ASD is not yet fully understood. Numerous studies have focused on the differences in intestinal microbial and metabolism profiles between typically developing (TD) children and children with ASD (10–14). It has been found that *Bifidobacterium*, *Blautia*, *Dialister*, *Prevotella*, *Veillonella*, and *Turicibacter* are decreased and that *Lactobacillus*, *Bacteroides*, *Desulfovibrio*, and *Clostridium* are increased in the gut microbiota of children with ASD relative to TD children (11). However, other inconsistent results of gut microbiota between TD and ASD children have also been reported (15). Current research on the metabolism of children with ASD mainly focuses on detection in plasma and feces. Plasma amino acid profiles, including glutamic acid, aspartic acid, and taurine, tend to be altered in ASD children (16). However, in both plasma (17) and feces (18), abnormal tryptophan metabolism has been reported in children with ASD. Meanwhile, a correlation between altered concentrations of tryptophan and serotonin and gut dysbiosis was observed in ASD patients (17). Further research has shown that differentially expressed microbes and metabolites between ASD and TD groups are involved in the regulation of neurotransmitter metabolic networks such as serotonin, dopamine, and gamma-aminobutyric acid (14), and it has been reported that serotonin may be associated with the symptoms of ASD (19). However, it is still not clear if these microbes and metabolites cause the development of ASD symptoms.

This study aimed to define the contribution of the gut microbiota to ASD etiology and explore the possible molecular mechanisms by which intestinal microorganisms influence behavioral changes in ASD. For this purpose, we transferred fecal microbiota from children with ASD and TD children into GF mice to determine whether ASD-relevant behaviors were transmissible via the gut microbiome. We performed 16S rRNA gene sequencing of the cecal contents of the mice as well as untargeted metabolomic analysis of cecal, serum, and prefrontal cortex (PFC) samples to identify differentially expressed gut microbial communities and altered metabolites and thereby discovered the metabolic pathways that were potentially influenced by the differentially expressed intestinal microbes. In addition, we performed untargeted metabolomic analysis of fecal samples obtained from TD children and children with ASD to verify the findings of the animal experiments. Finally, we measured the expression levels of key proteins in the colon and brain tissues of mice that had received fecal microbiota transplantation (FMT) from TD children and children with ASD and thus identified significantly enriched metabolic pathways and preliminarily explored the possible underlying molecular mechanisms.

## RESULTS

### FMT from children with ASD facilitated abnormal behaviors in GF mice.

**FIG 1:**
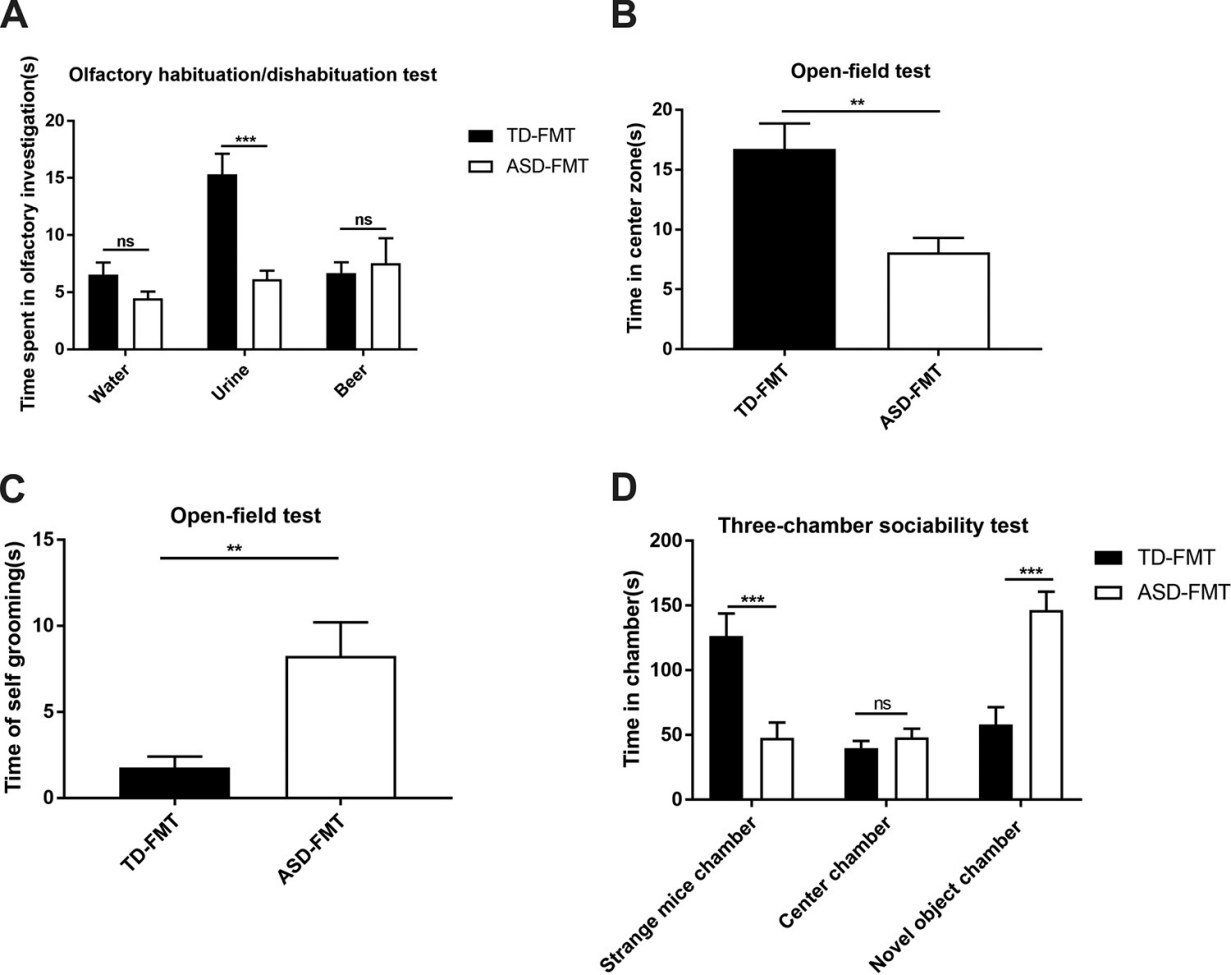
Behavioral tests on mice colonized with fecal samples from typically developing control (TD) donors and donors with autism spectrum disorder (ASD). (A) Olfactory habituation/dishabituation test: cumulative time spent sniffing cotton swabs. (B) Open-field test: comparison of times spent in the center zone between the two groups. (C) Open-field test: comparison of the self-grooming times between the two groups. (D) Three-chamber sociability test: time spent in different boxes in the two groups. Values are expressed as means ± SEM (*n* = 16). *, *P < *0.05; **, *P < *0.01; ***, *P < *0.001; ns, not significant (based on *post hoc* tests). TD-FMT, mice transplanted with the fecal microflora of TD donors; ASD-FMT, mice transplanted with the fecal microflora of donors with ASD.

To determine whether the gut microbiota of children with ASD are related to autism-like behaviors in mice, GF mice were transplanted with fecal samples collected from TD children (TD-FMT mice) and children diagnosed with ASD according to *Diagnostic and Statistical Manual of Mental Disorders*, 5th edition (DSM-5), criteria (ASD-FMT mice) (20). We performed behavioral tests on the mice at the 3rd week following FMT. The olfactory habituation/dishabituation test was conducted to compare the times spent sniffing water, mouse urine, and beer between the TD-FMT and ASD-FMT groups. The time spent sniffing mouse urine was significantly shorter in the ASD-FMT group than in the TD-FMT group (*P < *0.001) (Fig. 1A). In contrast, the times spent sniffing water and beer did not differ between the two groups, indicating that FMT did not affect the sense of smell (see Fig. S1 in the supplemental material) and suggesting that gut microbiota from children with ASD may affect sensitivity to social odors.

10.1128/mSystems.01343-20.1 FIG S1 Olfactory habituation/dishabituation test: comparison of cumulative times spent sniffing cotton swabs among the GF (germ-free), TD-FMT (mice transplanted with the fecal microflora of typically developing control donors), and ASD-FMT (mice transplanted with the fecal microflora of donors with autism spectrum disorder) groups. The values are expressed as the means ± SEM (*n* = 16). ***, *P < *0.001. Download FIG S1, DOC file, 0.10 MB. Copyright © 2021 Xiao et al. 2021 Xiao et al. https://creativecommons.org/licenses/by/4.0/ This content is distributed under the terms of the Creative Commons Attribution 4.0 International license.

The open-field test is a classic method to detect the spontaneous behaviors of mice in an unfamiliar environment, including anxiety and repetitive behaviors. During this test, the time of movement in the center zone was significantly shorter in the ASD-FMT group than in the TD-FMT group (Fig. 1B) (*P < *0.01). In contrast, the time of self-grooming was significantly longer in the ASD-FMT group than in the TD-FMT group (Fig. 1C) (*P < *0.01).

The three-chamber sociability test is designed to evaluate social behaviors in mice. This test showed that compared to the TD-FMT group, the ASD-FMT group displayed a significantly greater preference for the novel-object chamber (*P < *0.001) and a lesser preference for the strange-mouse chamber (*P < *0.001) (Fig. 1D). Furthermore, the mice in the ASD-FMT group showed increased repetitive behaviors and decreased sociability compared to the mice in the TD-FMT group. Taken together, these data suggest that the gut microbiota of children with ASD induced some core symptoms of ASD in the mice.

### FMT from children with ASD led to differential microbial community structures in GF mice.

**FIG 2:**
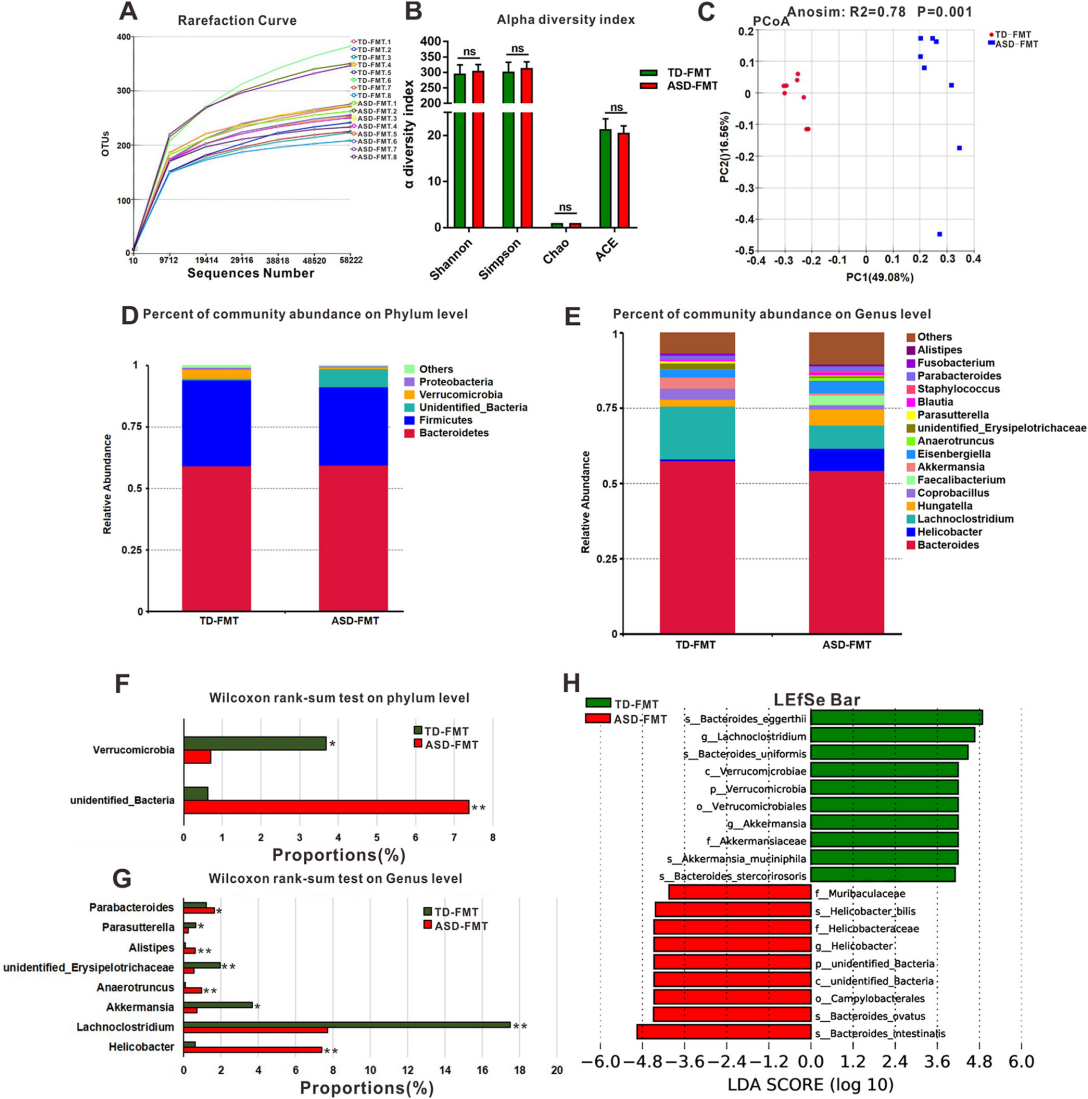
Comparison of microbial characteristics of cecal contents between the TD-FMT mice (mice transplanted with the fecal microflora of typically developing donors) and ASD-FMT mice (mice transplanted with the fecal microflora of donors with autism spectrum disorder). (A and B) Rarefaction curves (A) and alpha diversity indices (B) of cecal samples from TD-FMT and ASD-FMT mice. (C) Principal-coordinate analysis (PCoA) plots based on the unweighted UniFrac distance metrics of the cecal microbiota compositions between the two groups at the operational taxonomic unit (OTU) level. Anosim, analysis of similarity. (D and E) Percentages of community abundance in the TD-FMT and ASD-FMT mice at the phylum (D) and genus (E) levels. (F and G) Comparison of the dominant microflora at the phylum (F) and genus (G) levels between the TD-FMT and ASD-FMT mice according to the Wilcoxon rank sum test. (H) Histogram of the linear discriminant analysis (LDA) value distribution for the most abundant phylotypes of the cecal microbiota in the TD-FMT and ASD-FMT mice. *, *P < *0.05; **, *P < *0.01; ns, not significant (based on *post hoc* tests) (*n* = 8).

The rarefaction curves for all the cecal samples collected from the TD-FMT and ASD-FMT mice tended to approach the saturation plateau (Fig. 2A), suggesting that the sequencing depth was enough to cover the whole bacterial diversity and was suitable for the present study. In addition, no statistically significant differences were found in α diversity (Chao, abundance-based coverage estimator [ACE], Shannon, and Simpson diversity indices) between the two groups (Fig. 2B). Analysis of similarities showed that the differences between the two groups were significantly greater than the differences within the group (*R*^2^ = 0.78; *P* = 0.001) (Fig. 2C), indicating that our grouping was meaningful. Principal-coordinate analysis (PCoA) plots revealed conspicuous differences in microbial community structures between the TD-FMT and ASD-FMT groups (Fig. 2C). This finding shows that the mice in the TD-FMT and ASD-FMT groups formed microbial communities with distinct characteristics. The compositions of the dominant microflora in the TD-FMT and ASD-FMT groups at the phylum level are shown in Fig. 2D. *Bacteroidetes*, *Firmicutes*, *unidentified*_*Bacteria*, *Verrucomicrobia*, and *Proteobacteria* were the most abundant bacteria present in the cecum in the two groups. Further analysis revealed that the abundance of *Verrucomicrobia* was significantly lower in the ASD-FMT group than in the TD-FMT group (Fig. 2F) (*P < *0.05), while that of *unidentified*_*Bacteria* was higher in the ASD-FMT group than in the TD-FMT group (Fig. 2F) (*P < *0.01). The predominant genera in the TD-FMT and ASD-FMT groups are shown in Fig. 2E. At the genus level, *Parabacteroides* (*P < *0.05), *Alistipes* (*P < *0.01), *Anaerotruncus* (*P < *0.01), and *Helicobacter* (*P < *0.01) were increased in the ASD-FMT group compared with the TD-FMT group, while *Parasutterella* (*P < *0.05), *Akkermansia* (*P < *0.05), *Lachnoclostridium* (*P < *0.01), and *unidentified*_*Erysipelotrichaceae* (*P < *0.01) were decreased in the ASD-FMT group, according to the Wilcoxon rank sum test (Fig. 2G). To further identify the key microbiota members responsible for discriminating TD-FMT mice from ASD-FMT mice, we used the linear discriminant analysis (LDA) effect size (LEfSe) method. *Verrucomicrobia* and *unidentified*_*Bacteria* showed statistically significant differences between the two groups and were identified as key phyla. At the genus level, *Helicobacter*, *Lachnoclostridium*, and *Akkermansia* showed significant between-group differences and were identified as key genera (Fig. 2H). The above-described data suggest that the microbial community in the cecum of the mice has different characteristics after FMT.

### FMT altered the tryptophan metabolism and serotonergic synapse pathways in mice.

**FIG 3:**
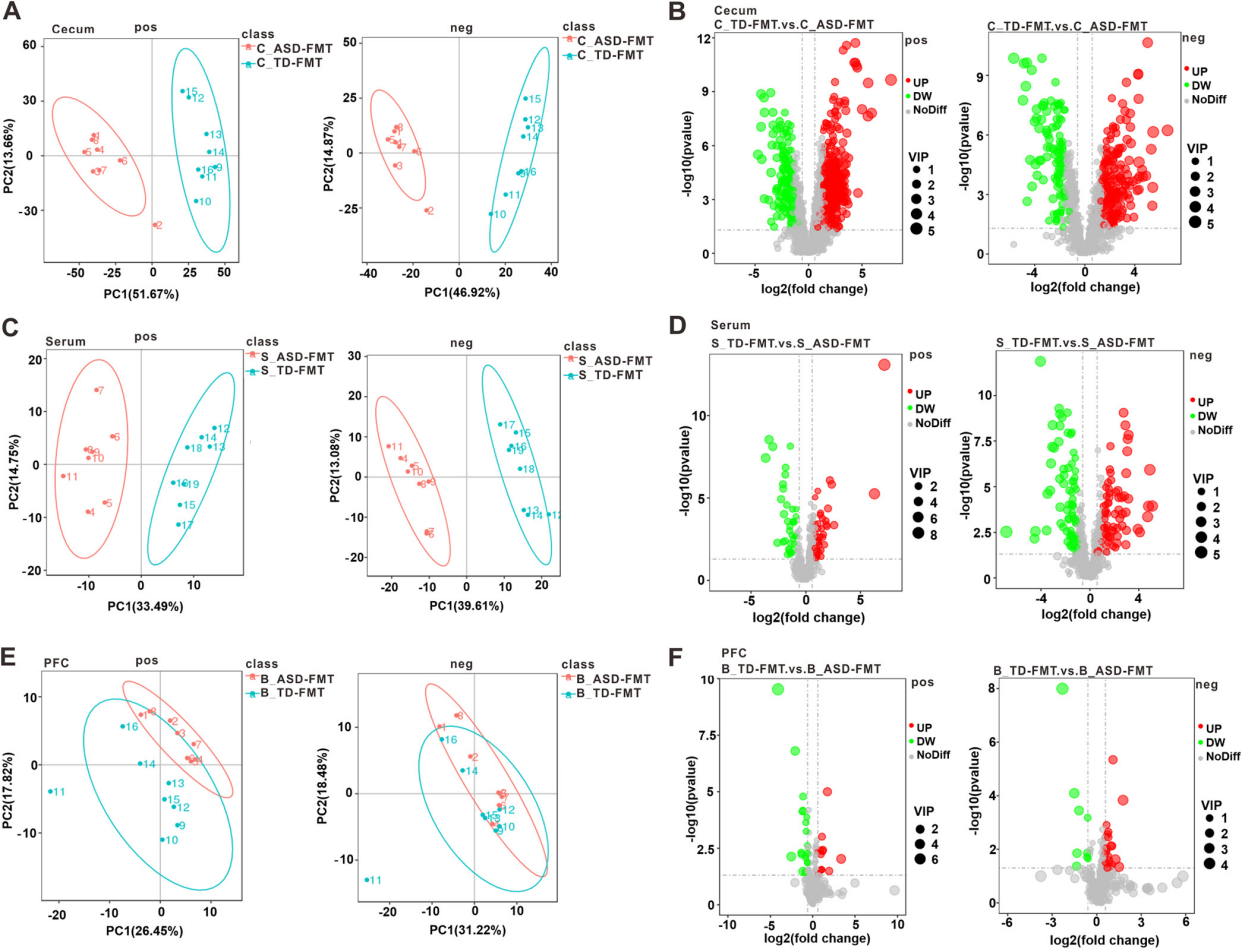
Differentially abundant metabolites identified by untargeted metabolomics in different tissues of TD-FMT mice (mice transplanted with the fecal microflora of typically developing control donors) and ASD-FMT mice (mice transplanted with the fecal microflora of donors with autism spectrum disorder). (A, C, and E) Principal-component analysis (PCA) of the cecal contents (A), serum samples (C), and prefrontal cortex (PFC) samples (E) between the TD-FMT and ASD-FMT groups. (B, D, and F) Volcano plots of the altered metabolites in the cecal contents (B), serum samples (D), and PFC samples (F) between the two groups (*n* = 8). The abscissa represents the log_2_(fold change) of metabolite expression, and the ordinate represents the level of the −log_10_(*P* value). VIP, variable importance in the projection; pos, positive-ion mode; neg, negative-ion mode; UP, the upregulated metabolites in ASD-FMT group compared with TD-FMT group; DW, the downregulated metabolites in ASD-FMT group compared with TD-FMT group; NoDiff, the metabolites with no difference between TD-FMT and ASD-FMT groups.

We performed untargeted metabolomics analyses of cecal contents, serum samples, and prefrontal cortex samples collected from the TD-FMT and ASD-FMT mice in order to identify the metabolic pathways of the intestinal microbiome that are involved in ASD. Liquid chromatography-tandem mass spectrometry (LC-MS/MS) analyses of the above-described samples were conducted in both the positive- and negative ion-modes. The cecal contents, serum samples, and prefrontal cortex samples from the TD-FMT and ASD-FMT groups were largely separated according to principal-component analysis (PCA) (Fig. 3A, C, and E). We identified 963 metabolites in the cecal contents that were differentially expressed between the TD-FMT and ASD-FMT groups, including 698 metabolites that were downregulated and 265 metabolites that were upregulated in the ASD-FMT group relative to the TD-FMT group (Fig. 3B). Similarly, a total of 239 differentially expressed metabolites were detected in the serum samples, including 122 downregulated metabolites and 117 upregulated metabolites in the ASD-FMT group (Fig. 3D). In the prefrontal cortex samples, 65 differentially expressed metabolites were identified, of which 36 were downregulated and 29 were upregulated in the ASD-FMT group (Fig. 3F).

**FIG 4:**
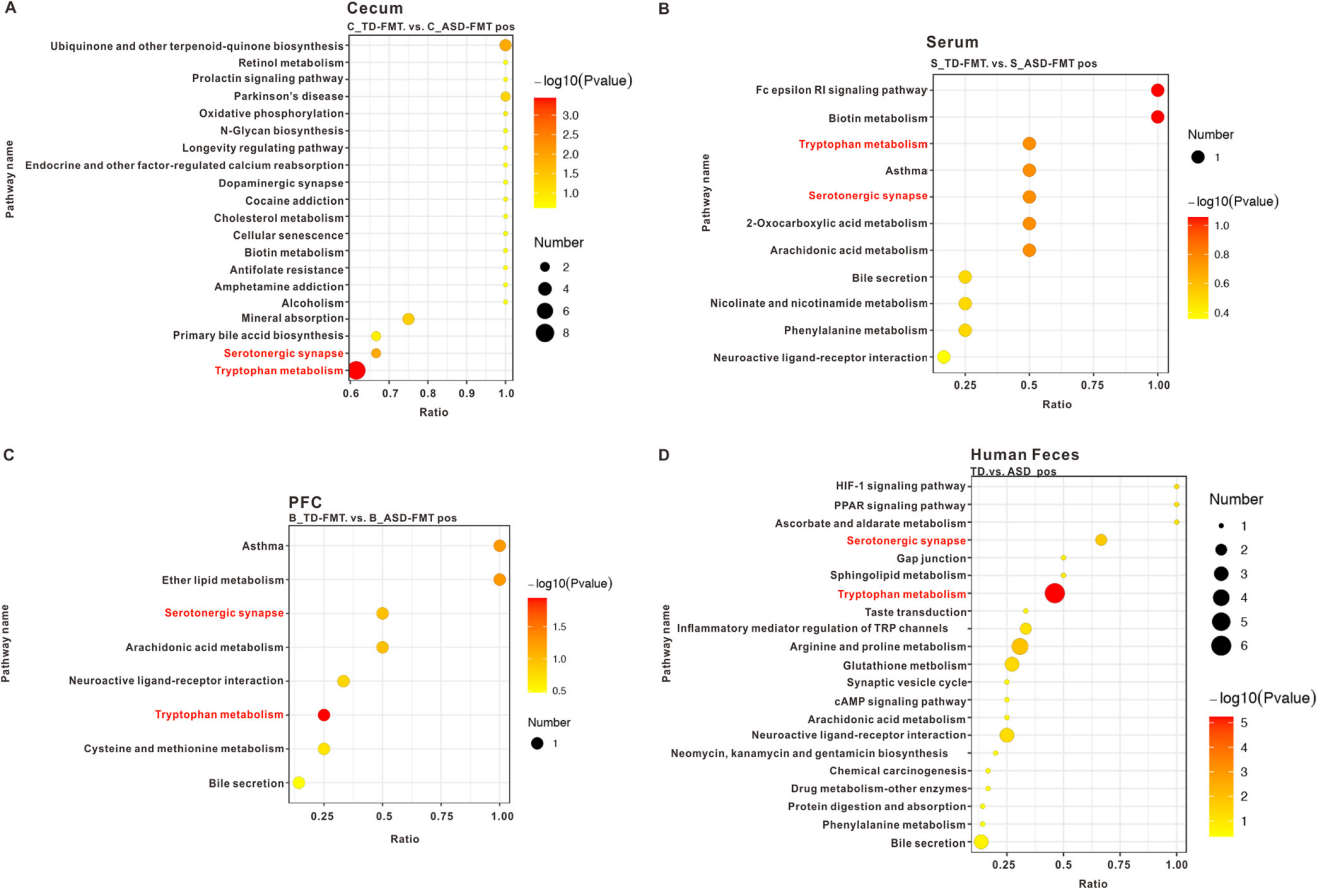
Metabolite enrichment analysis highlighted altered KEGG (Kyoto Encyclopedia of Genes and Genomes) pathways. (A to C) Differential KEGG pathways in the cecal contents (A), serum samples (B), and prefrontal cortex (PFC) samples (C) in the TD-FMT mice (mice transplanted with the fecal microflora of typically developing control donors) and ASD-FMT mice (mice transplanted with the fecal microflora of donors with autism spectrum disorder) (*n* = 8). (D) Differential KEGG pathways in the feces of TD children (*n* = 60) and children with ASD (*n* = 120). PPAR, peroxisome proliferator-activated receptor.

To demonstrate the biological functions of the differentially expressed metabolites, we performed Kyoto Encyclopedia of Genes and Genomes (KEGG) pathway enrichment analysis. The differentially expressed metabolites in the cecal contents of the TD-FMT and ASD-FMT mice were significantly enriched in some metabolic pathways, including retinol, cholesterol, and biotin (Fig. 4A). We found significant between-group differences in the enrichment of the biotin metabolism pathway in the cases of both the cecal contents and serum samples (Fig. 4A and B). The differential metabolites in the prefrontal cortex and serum were enriched in the pathway related to bile secretion (Fig. 4B and C). Interestingly, our study found that the differential metabolites in the cecal contents, serum samples, and prefrontal cortex samples were enriched in the tryptophan metabolism and serotonergic synapse pathways (Fig. 4A to C).

### Tryptophan metabolism and serotonergic synapse pathways were also altered in fecal samples of children with ASD.

**FIG 5:**
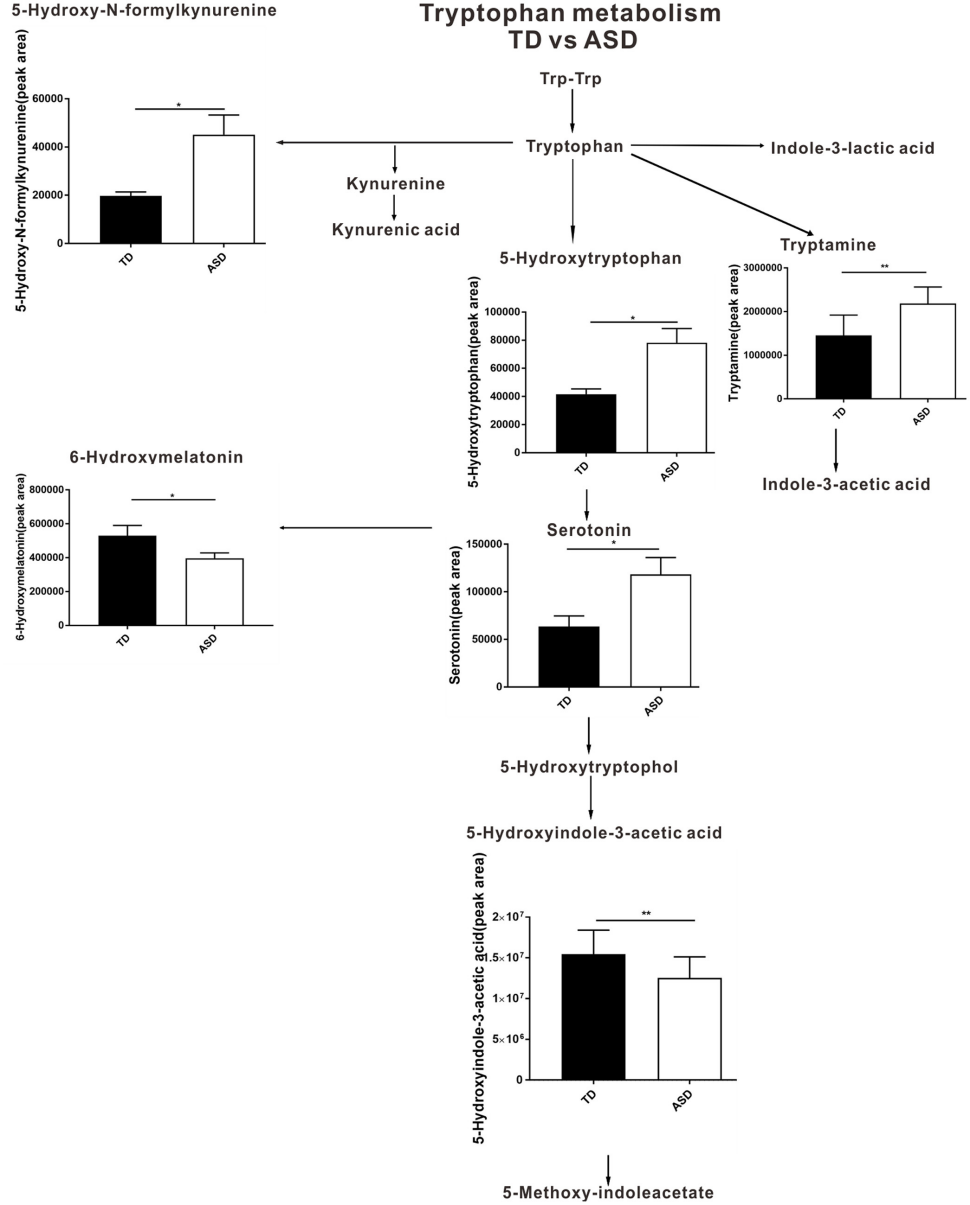
Changed metabolites in the tryptophan and serotonin metabolic pathways between TD (typically developing control) (*n* = 60) and ASD (autism spectrum disorder) children (*n* = 120). Differential metabolites were screened according to a fold change of >1.25 or <0.8 and a *P* value of <0.05 (by a *t* test). *, *P < *0.05; **, *P < *0.01.

**FIG 6:**
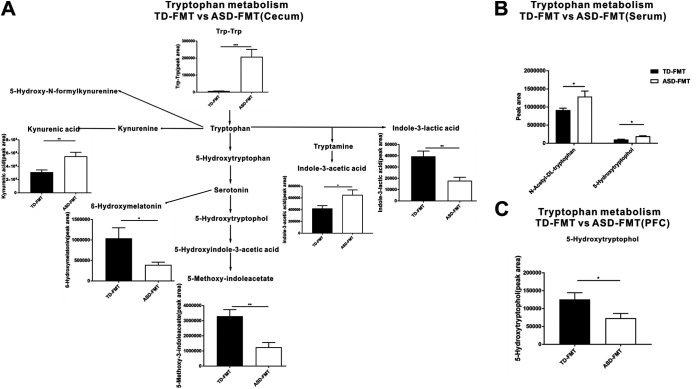
Differential metabolites in the tryptophan and serotonin metabolic pathways at different tissue sites between the TD-FMT (mice transplanted with the fecal microflora of typically developing control donors) and ASD-FMT (mice transplanted with the fecal microflora of donors with autism spectrum disorder) groups (*n* = 8). Shown are changed metabolites in the tryptophan and serotonin metabolic pathways in the cecum (A), serum (B), and prefrontal cortex (PFC) (C) between the TD-FMT and ASD-FMT groups. Differential metabolites were screened according to a fold change of >1.25 or <0.8 and a *P* value of <0.05 (by a *t* test). *, *P < *0.05; **, *P < *0.01; ***, *P < *0.001.

To validate the results obtained in the animal experiments, we recruited 60 TD children and 120 children with ASD to participate in our study. Except for picky eating behavior (TD, 26/60 [43.3%]; ASD, 80/120 [66.7%] [*P < *0.001]), the TD children were age, gender, region, height, weight, and socioeconomic status matched with the ASD group (data not shown). Fecal metabolites in both groups of children were analyzed using untargeted metabolomics. Interestingly, the metabolite enrichment analysis of the fecal samples of the children also highlighted the tryptophan metabolism and serotonergic synapse KEGG pathways (Fig. 4D). To further explore the specific differential metabolites in the tryptophan metabolism and serotonergic synapse pathways, we mapped a brief metabolic pathway for tryptophan and serotonin. We found that 5-hydroxy-*N*-formylkynurenine, tryptamine, 5-hydroxytryptophan, and serotonin were more abundant in children with ASD than in TD children and that 6-hydroxymelatonin and 5-hydroxyindole-3-acetic acid were decreased in the children with ASD relative to the TD children (Fig. 5). In addition, in the cecal contents of mice, the levels of Trp-Trp (a dipeptide of tryptophan), kynurenic acid, and indole-3-acetic acid were significantly higher and those of 6-hydroxymelatonin, indole-3-lactic acid, and 5-methoxy-indoleacetate were significantly lower in the ASD-FMT group than in the TD-FMT group (Fig. 6A). Furthermore, the *N*-acetyl-dl-tryptophan and 5-hydroxytryptophol levels were significantly increased in the serum samples, but 5-hydroxytryptophol was significantly decreased in the prefrontal cortex samples of the ASD-FMT mice relative to the TD-FMT mice (Fig. 6B and C). Although the differentially enriched fecal metabolites in the tryptophan metabolism and serotonin synapse pathways were not identical in the human and animal experiments, our findings show that ASD-related intestinal microbes induced abnormal tryptophan and serotonin metabolism in both ASD-FMT mice and children with ASD.

### Specific bacteria in mice after FMT may be associated with tryptophan and serotonin metabolism.

**FIG 7:**
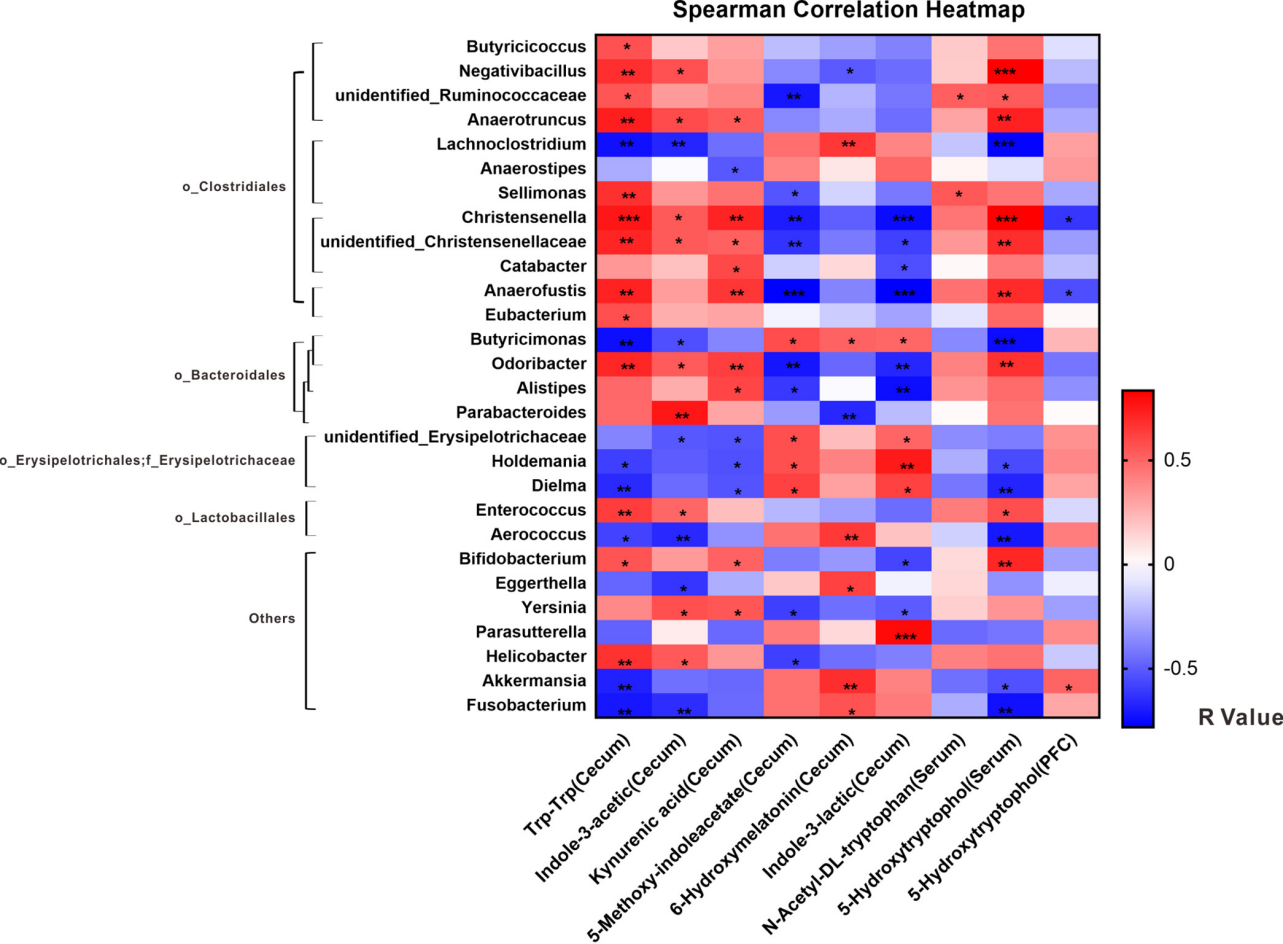
Correlations of significantly different metabolites in the tryptophan and serotonin metabolic pathways with genera in the TD-FMT (mice transplanted with the fecal microflora of typically developing control donors) and ASD-FMT (mice transplanted with the fecal microflora of donors with autism spectrum disorder) groups. The correlation effect is indicated by a color gradient from blue (negative correlation) to red (positive correlation) (*n* = 8). *, *P < *0.05; **, *P < *0.01; ***, *P < *0.001.

To explore the potential relationships between the gut microbiota and metabolites, we created a correlation matrix by using Spearman correlation. The correlations among the differentially expressed metabolites and microbiota in the cecal contents in the TD-FMT and ASD-FMT groups are shown in Fig. 7. *Parabacteroides*, *Alistipes*, *Anaerotruncus*, *Helicobacter*, *Parasutterella*, *Akkermansia*, *Lachnoclostridium*, and *unidentified*_*Erysipelotrichaceae* were identified as genera with significant differences between the ASD-FMT and TD-FMT groups (Fig. 2G) and were related to certain differentially expressed metabolites involved in tryptophan and serotonin metabolism. Spearman correlation (Fig. 7) showed that the upregulated metabolites in the cecal contents from the ASD-FMT group, including Trp-Trp, kynurenic acid, and indole-3-acetic acid, were positively correlated with the majority of the genera in the *order_Clostridiales* (*o_Clostridiales*), except for *Lachnoclostridium* and *Anaerostipes*. Furthermore, these three significantly upregulated metabolites in the ASD-FMT group were positively correlated with some genera in the *order_Bacteroidetes* (*o_Bacteroidetes*) (but not *Butyricimonas*) and negatively correlated with the *order_Erysipelotrichales* (*o_Erysipelotrichales*), *family_Erysipelotrichaceae* (*f_Erysipelotrichaceae*). Surprisingly, almost all the genera that were positively correlated with the metabolites upregulated in the ASD-FMT group were negatively correlated with the downregulated metabolites and vice versa. These results further confirmed that some specific bacteria may be associated with tryptophan and serotonin metabolism, including some genera belonging to the *o_Clostridiales*, *o_Bacteroidetes*, and *f_Erysipelotrichaceae*.

### FMT from ASD donors altered key proteins involved in serotonin synthesis and transport in colonized mice.

**FIG 8:**
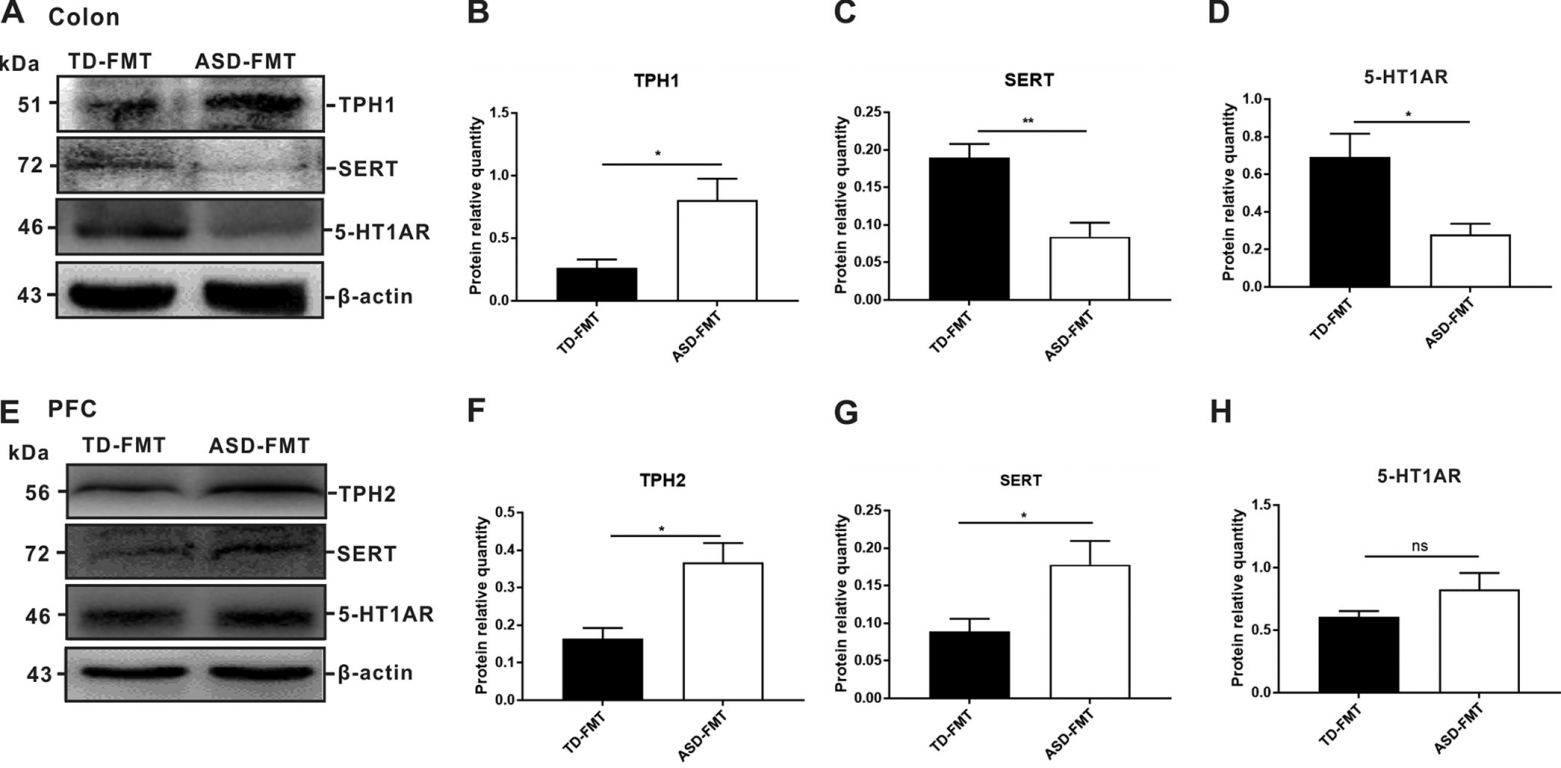
(A) Protein expression levels of TPH1 (tryptophan hydroxylase), SERT (serotonin transporter), and 5-HT1AR (serotonin 1A receptor) in the colons of the TD-FMT (mice transplanted with the fecal microflora of typically developing control donors) and ASD-FMT (mice transplanted with the fecal microflora of donors with autism spectrum disorder) groups. (E) Protein expression levels of TPH2, SERT, and 5-HT1AR in the prefrontal cortex (PFC). (B to D) Western blot (WB) signal quantification for TPH1 (B), SERT (C), and 5-HT1AR (D) in the colon. (F to H) WB signal quantification for TPH2 (F), SERT (G), and 5-HT1AR (H) in the PFC. Values are expressed as means ± SEM (*n* = 3). *, *P < *0.05; **, *P < *0.01; ns, not significant (based on *post hoc* tests).

Tryptophan is a precursor of serotonin. Our study found that the gut microbiota from children with ASD influenced tryptophan and serotonin metabolism in GF mice after FMT. To further investigate the possible mechanisms underlying this finding, we examined the protein expression levels of tryptophan hydroxylase (TPH) (a key enzyme in the gastrointestinal synthesis of serotonin), serotonin transporter (SERT), and serotonin 1A receptor (5-HT1AR) in the colon and prefrontal cortex. The protein expression level of TPH1 in the colon was notably upregulated in the ASD-FMT group compared with the TD-FMT group (*P < *0.05) (Fig. 8A and B). The expression levels of both SERT (*P < *0.01) and 5-HT1AR (*P < *0.05) in the colon were downregulated in the ASD-FMT group compared with the TD-FMT group (Fig. 8A, C, and D). In the prefrontal cortex, TPH2 is the key enzyme involved in serotonin synthesis. The expression levels of TPH2 and SERT in the prefrontal cortex were significantly upregulated in the ASD-FMT group compared with the TD-FMT group (*P < *0.05 for both) (Fig. 8E to G). The protein expression levels of 5-HT1AR in the prefrontal cortex did not differ between the two groups (*P > *0.05) (Fig. 8H). Considering these data, we hypothesized that the gut microbiota from donors with ASD resulted in abnormalities in tryptophan and serotonin metabolism and altered the key proteins involved in the synthesis and transport of serotonin in mice.

## DISCUSSION

The fecal microbiota can influence brain function and behaviors through the microbiota-gut-brain axis and may contribute to a variety of neuropsychiatric disorders, including depression, schizophrenia, Parkinson’s disease, and autism (21–24). It has been reported that GF mice colonized with the gut microbiota of patients with neurodevelopmental disorders develop disease-relevant behavioral phenotypes (24), which provides a basis for studying the interaction of gut microbes and ASD. Sandler et al. speculated that enteric dysbacteriosis may lead to intestinal colonization by neurotoxin-producing bacteria, which is related to ASD symptoms (25). Since then, numerous studies have reported differences in fecal microorganisms between TD children and children with ASD, especially children with ASD and gastrointestinal symptoms (11, 26, 27). Furthermore, microbiota transfer therapy, which changes the intestinal microenvironment, has improved behavioral problems and gastrointestinal symptoms in children with ASD (28). These findings highlight a possible link between gut microbes and ASD behaviors. Our experiments showed that GF mice colonized with microbiota from children with ASD displayed ASD-relevant behaviors, including increased repetitive behaviors and decreased sociability, which further supports the possibility that fecal microbiota from children with ASD may promote ASD-relevant behaviors in GF mice.

Several studies have noted that children with ASD have different microbial community structures and metabolic profiles relative to TD children (11–13). The present study revealed that gut microbiota from children with ASD affected the distribution and composition of intestinal microorganisms in GF mice. *Akkermansia* was decreased in the ASD-FMT mice in our study. A lower abundance of *Akkermansia* in children with ASD has been reported to indicate a thinner gastrointestinal mucus barrier (29). Butyric acid is known to promote neuroplasticity and memory formation (30), and *Clostridiales* and *Erysipelotrichaceae* have been reported to be associated with the production of butyric acid (31). In our study, *Lachnoclostridium*, which belongs to the *o_Clostridiales*, and *Erysipelotrichaceae* were highly decreased in the ASD-FMT group. Tryptophan is the only precursor of serotonin, and most of the serotonin produced from tryptophan is stored in chromaffin cells and released in response to various stimuli in the gut lumen (17, 32). More than 90% of the total tryptophan is oxidized via the kynurenine pathway into kynurenic acid or via the quinolinic acid pathway into quinolinic acid (33). Tryptophan can also be metabolized by the gut microbiota into indole and its derivatives (34). Patients with ASD exhibit altered tryptophan metabolism, with reduced levels of tryptophan and an increased kynurenine-to-tryptophan ratio in the plasma (35). Despite contrary findings, most reports still support the conclusion that in autistic children, the serotonin level is increased in the serum and decreased in the brain (36). Animal studies have confirmed that tryptophan and serotonin metabolism is regulated by gut microbes (37). Our study showed that in the cecum, compared with the TD-FMT mice, the ASD-FMT mice showed increased levels of the kynurenine pathway products involved in tryptophan metabolism, decreased levels of serotonin metabolism products, and altered indole products involved in tryptophan metabolism. Surprisingly, increased levels of the kynurenine pathway products and decreased levels of serotonin metabolism products were also obtained in ASD children compared with TD children. Therefore, our study suggests that changes in tryptophan and serotonin metabolism may be the result of altered intestinal microorganisms.

Recent studies have revealed a significant increase in several mucosa-associated members of the *Clostridiales* in patients with ASD (38). Indigenous spore-forming bacteria, including *Clostridiales*, promote serotonin biosynthesis from colonic enterochromaffin cells (39). In particular, some bacteria belonging to the genera *Clostridium*, *Ruminococcus*, *Blautia*, and *Lactobacillus* have been identified as being able to convert tryptophan to tryptamine (40). Consistent with the above-described results, our study suggested that most of the genera of the *Clostridiales* were positively correlated with increased tryptophan and serotonin metabolites in the cecum of ASD-FMT mice and negatively correlated with decreased metabolites of tryptophan and serotonin. Short-chain fatty acids are the main metabolites produced by bacteria fermenting dietary fiber in the gastrointestinal tract, including acetic acid, propionic acid, isobutyric acid, butyric acid, isopentyl acid, and pentanoic acid (41). Propionic acid may induce ASD-like behaviors upon intraventricular administration, and the underlying mechanism may be related to the regulation of neurotransmitters (42, 43). *Clostridia* and *Bacteroides* can produce propionic acid (44). In the current study, *o_Bacteroidales* were found to be involved in tryptophan and serotonin metabolism. Therefore, we speculated that some genera belonging to the *o_Bacteroidales* may be related to the production of propionic acid and may affect ASD behaviors by regulating tryptophan and serotonin metabolism. Liu et al. reported that butyrate-producing taxa belonging to the *Erysipelotrichaceae* were significantly decreased in the feces of individuals with ASD (12). Our study not only supported this conclusion but also further revealed that genera belonging to the *f_Erysipelotrichaceae*, including *unidentified*_*Erysipelotrichaceae*, *Holdemania*, and *Dielma*, were involved in tryptophan and serotonin metabolism in ASD-FMT mice.

TPH catalyzes the rate-limiting step in serotonin synthesis (45). TPH1 is present mainly in peripheral organs such as the intestines and spleen, while TPH2 predominates in the brain stem (46). High peripheral serotonin levels are associated with changes in SERTs and serotonin receptors in children with ASD (47). SERTs are located on presynaptic serotonin nerve terminals as well as on axons and serotonin cell bodies in the raphe (48). These transporter molecules can rapidly clear serotonin from the synaptic gap and effectively manage serotonin concentrations; thus, alterations in SERT levels can lead to physiological dysfunction (49). The 5-HT1A receptor is predominant in the prefrontal cortex and regulates the release of serotonin through a negative-feedback loop. Studies have shown that the number of postsynaptic 5-HT1A receptors is decreased in the brains of patients with anxiety and depression (50). In the present study, the colonic expression level of TPH1 was higher and the expression levels of SERT and 5-HT1AR were lower in the ASD-FMT mice than in the TD-FMT mice. These results may be attributable to self-regulation caused by decreased serotonin metabolites in ASD-FMT mice. Studies have shown that in children with ASD, the serotonin level is increased in the serum and decreased in the brain (36). Our results revealed that TPH2 and SERT expression levels were significantly increased in the prefrontal cortex of the ASD-FMT mice relative to the TD-FMT mice. We therefore speculate that the increased SERT expression in the prefrontal cortex of the ASD-FMT mice may lead to a decrease in the central serotonin level. Overall, the above-described results provide preliminary clues about the possible molecular mechanisms by which intestinal microorganisms influence tryptophan and serotonin metabolism.

There are some limitations of our study. First, it is difficult to distinguish whether the metabolites in the mouse cecum were derived from the host or from the gut microbiota. Second, the causal relationship between gut microbes, metabolites, and key metabolic proteins involved remains unclear. Finally, multiple factors can affect the gut microbiome and metabolomics, and we cannot fully control the potential impacting factors. Therefore, further research should focus on controlling for confounders; verifying the causal relationship between specific intestinal microorganisms, metabolites, and ASD in animals or in *in vitro* experiments; and elucidating the underlying molecular mechanisms.

### Conclusions.

The fecal microbiome from children with ASD led to ASD-like behaviors, distinct microbial community structures, and altered tryptophan and serotonin metabolism in GF mice. The tryptophan and serotonin metabolism pathways were altered in both children with ASD and TD children and in ASD-FMT and TD-FMT mice. Some specific microbiota, namely, *o_Clostridiales*, *o_Bacteroidales*, and *f_Erysipelotrichaceae*, were related to tryptophan and serotonin metabolism. In addition, ASD-related intestinal microorganisms may alter the expressions of related proteins involved in serotonin synthesis and transport in GF mice, which may be the molecular basis for the changes in tryptophan and serotonin metabolism. This study provides valuable clues for studying the relationship between intestinal microorganisms and ASD. However, the etiology and specific regulatory mechanisms remain to be elucidated. Future studies are urgently needed to further validate our findings in animals and human cohorts, confirm the effects of certain microorganisms and metabolisms on ASD, and elucidate the underlying regulatory mechanisms.

## MATERIALS AND METHODS

### Animals, donor selection, and FMT.

This study was approved by the ethics committee of Chongqing Medical University (Chongqing, China) and the Army Medical University (Chongqing, China). Thirty-two male GF BALB/c mice were obtained from the Department of Laboratory Animal Science of the Army Medical University (Chongqing, China) and bred in a gnotobiotic environment. The mice were housed in a single room with constant temperature (20°C to 23°C) and humidity (40% to 70%). Autoclaved chow and water were provided *ad libitum*, and a 12-h light/12-h dark cycle was maintained.

In this study, 5 TD children and 5 ASD children were recruited, and their fecal samples were collected as donor samples. The diagnosis of ASD was made by a developmental pediatrician or psychologist through a series of structured interviews, according to DSM-5 criteria. Health examinations were also conducted for the TD donors to confirm that they did not have any signs of developmental disorders or psychiatric diseases. All 10 donors were boys aged 2 to 6 years with no history of other developmental disorders, neurological or psychiatric diseases, genetic metabolic diseases, or major physical illness. In addition, none of the donors had received any medications, antibiotics, antifungals, probiotics, or prebiotics in the past 3 months. The participants volunteered to participate in the study as fecal donors, and their parents signed an informed consent form. The study protocol was approved by the institutional review board of Children’s Hospital, Chongqing Medical University. This clinical trial was registered in the Chinese Clinical Trial Registry (registration number ChiCTR-ROC-14005442). Fresh fecal samples from the ASD and TD donors were collected in clean collection tubes and rapidly frozen at −80°C. Fecal samples of equal quantities from the 5 TD donors were thoroughly mixed with phosphate buffer (15 ml/g of feces), and the fecal residue was removed (51). The same fecal suspension method was used to prepare the fecal samples from the ASD group.

GF mice aged 4 weeks were randomly divided into 2 groups: the mice in the TD-FMT group were gavaged with 200 μl of the fecal suspension obtained from the TD children, and the mice in the ASD-FMT group were gavaged with the same volume of the fecal suspension obtained from the children with ASD. After colonization, the mice were maintained under the same feeding conditions as the ones described above. The 2 groups of mice were raised in 2 separate, gnotobiotic environments. After 3 weeks, the mice were used for subsequent experiments.

### Behavioral testing.

At the age of 7 weeks, all the mice were subjected to the following tests in the order given: olfactory habituation/dishabituation test, open-field test, and three-chamber sociability test. To ensure the quality of the experiment, the mice were given 1 day off after each type of test was completed. All the behavioral tests were conducted in a quiet and gentle environment. The test boxes or cages were cleaned of urine and feces and sterilized with 75% alcohol before the next mouse was tested. The mice were put back into their experimental environments immediately after the test was completed.

### (i) Olfactory habituation/dishabituation test.

Urine was collected in advance from adult BALB/c males. Each test animal was habituated to the clean empty cage for 10 min before the experiment began. Next, fluid-filled swabs were suspended 10 cm from the bottom of the box to ensure that the mouse could smell them. Each mouse was required to sniff 3 types of cotton swabs containing water, mouse urine, and beer in turn (52). The observation time for each cotton swab was 3 min, and the total time for each cotton swab was recorded. This procedure was performed twice, with a 10-min rest period between every two experiments. The average times for the 3 types of cotton swabs were recorded.

### (ii) Open-field test.

The open-field test was carried out as described previously (9, 53). Mice were habituated in the testing room and left undisturbed for 30 min prior to testing. Each mouse was placed in the same position in the box at the beginning of the experiment. Data were collected from each mouse placed in the plexiglass open-field arena (40 cm [length] by 40 cm [width] by 30 cm [height]) for a period of 5 min. ANY-maze software and the supporting camera system automatically recorded the total distance traveled, the number of lines crossed, the time spent in the center zone, and the self-grooming time for each subject.

### (iii) Three-chamber sociability test.

The three-chamber sociability test was performed, as described previously (54), in a 60- by 40-cm^2^ white plexiglass box divided into 3 chambers (20 by 40 cm^2^) by plexiglass dividers. The mice could freely move through a small opening (6 by 6 cm) in each divider and were observed for 5 min in the empty box to confirm that each animal had no bias for any of the chambers. A sex- and age-matched adult SPF BALB/c mouse was placed in a small cage in one chamber (strange-mouse chamber), while a small object was placed in another chamber (novel-object chamber) in advance. The mouse being tested was placed in the center chamber and allowed to travel between the chambers for 5 min, while an overhead camera recorded its movements. The time spent in each of the 3 chambers was recorded.

### Tissue and sample collection.

After the behavioral experiments, the mice were sacrificed using an intraperitoneal injection of sodium pentobarbital. Blood was immediately collected from the eyeball, and the separated serum was frozen at −80°C until analysis. The prefrontal cortex was frozen at −80°C immediately after the macrocutting of the brain for subsequent experiments. The cecum and colon were extracted, put into sample collection tubes separately, and frozen at −80°C for further study.

### 16S rRNA gene sequencing.

Total genomic DNA was extracted from the cecum by using a magnetic soil and stool DNA kit (catalog number DP712; Tiangen, China) according to the manufacturer’s protocol. DNA concentration and purity were monitored on 1% agarose gels. According to the concentration, DNA was diluted to 1 ng/μl using sterile water. The V4 regions of the bacterial 16S rRNA gene were amplified with the barcoded primers 515F and 806R. All PCR assays were carried out in a 30-μl reaction volume with 15 μl Phusion high-fidelity PCR master mix (New England BioLabs), 0.2 μM the forward and reverse primers, and approximately 10 ng template DNA. Thermal cycling consisted of an initial denaturation step at 98°C for 1 min, followed by 30 cycles of denaturation at 98°C for 10 s, annealing at 50°C for 30 s, and elongation at 72°C for 30 s, and a final extension step at 72°C for 5 min. Equal volumes of 1× loading buffer (containing SYBR green) and PCR products were mixed and subjected to electrophoresis on 2% agarose gels. The PCR products were mixed in equidense ratios, and the mixture was purified using the GeneJET gel extraction kit (Thermo Scientific). Sequencing libraries were generated using Ion Plus fragment library kit 48 rxns (Thermo Scientific) according to the manufacturer’s recommendations. The library quality was assessed using the Qubit@2.0 fluorometer (Thermo Scientific). Finally, the library was sequenced on an Ion S5 XL platform, and 400-bp/600-bp single-end reads were generated.

### Microbial analysis.

Single-end reads were assigned to samples based on their unique barcode and truncated by cutting off the barcode and primer sequence. Quality filtering on the raw reads was performed under specific filtering conditions to obtain high-quality clean reads according to the Cutadapt (55) (V1.9.1 [http://cutadapt.readthedocs.io/en/stable/]) quality-controlled process. The reads were compared with the reference database (56) (Silva database [https://www.arb-silva.de/]) using the UCHIME algorithm (57) (http://www.drive5.com/usearch/manual/uchime_algo.html) to detect the chimera sequences, and the chimeras were removed (58). The clean reads were then finally obtained.

Uparse software (59) (v7.0.1001 [http://www.drive5.com/uparse/]) was used for sequence analysis. Sequences with ≥97% similarity were assigned to the same operational taxonomic units (OTUs), and the sequence with the highest frequency in the OTUs was selected as the representative sequence of the OTUs according to the algorithm principle. For each representative sequence, the Silva database (56) (https://www.arb-silva.de/) was used based on the Mothur (60) algorithm to annotate taxonomic information. Next, taxonomic information was obtained, and the community composition of each sample was determined at each taxonomic level. In order to study the phylogenetic relationship of different OTUs and the difference of the dominant species in different samples (groups), multiple-sequence alignments were conducted using MUSCLE software (61) (version 3.8.31 [http://www.drive5.com/muscle/]). Finally, OTU abundance information was normalized using a standard sequence number corresponding to the sample with the fewest sequences, and the output-normalized data were used for subsequent analysis.

Alpha diversity was applied for analyzing the complexity of species diversity for a sample through 4 indices, Chao (http://www.mothur.org/wiki/Chao), Shannon (http://www.mothur.org/wiki/Shannon), Simpson (http://www.mothur.org/wiki/Simpson), and ACE (http://www.mothur.org/wiki/Ace). All these indices in our samples were calculated with QIIME (version 1.7.0) (62) and displayed with R software (version 2.15.3).

The β-diversity was estimated by computing unweighted UniFrac distances and visualized using principal-coordinate analysis (PCoA), and PCoA analysis was displayed by using the WGCNA package, stat packages, and the ggplot2 package in R software (version 2.15.3). In addition, LEfSe was determined by using LEfSe software (LEfSe 1.0) to determine the community or species that influenced the group division the most (63). The nonparametric factorial Kruskal-Wallis sum rank test and LDA were performed to determine whether these features were consistent with the expected behaviors of the different bacterial taxonomic levels. Genera with LDA scores of >4 were defined as having a significant impact on the group.

### Metabolomic analyses of cecal contents, serum, and prefrontal cortex.

Cecal contents, serum samples, and prefrontal cortex samples were individually ground with liquid nitrogen, and the homogenates were resuspended in prechilled 80% methanol and 0.1% formic acid. The supernatants were collected after centrifugation (15,000 × *g* for 35 min at 4°C) and diluted to a final concentration of 60% methanol in liquid chromatography-mass spectrometry-grade water. The samples were subsequently transferred to a fresh Eppendorf tube with a 0.22-μm filter and centrifuged again at 15,000 × *g* for 10 min at 4°C. Finally, the filtrate was injected into an LC-MS/MS system for analysis.

LC-MS/MS analyses were performed using a Vanquish ultrahigh-performance liquid chromatography (UHPLC) system (Thermo Fisher) coupled with an Orbitrap Q Exactive series mass spectrometer (Thermo Fisher). Samples were injected onto a Hyperil gold column (100 by 2.1 mm, 1.9 μm) using a 16-min linear gradient at a flow rate of 0.2 ml/min. The Q Exactive series mass spectrometer was operated in the positive/negative-polarity mode with a spray voltage of 3.2 kV, a capillary temperature of 320°C, a sheath gas flow rate of 35 arbitrary units, and an auxiliary gas flow rate of 10 arbitrary units.

The raw data files generated by the UHPLC-MS/MS system were processed using Compound Discoverer 3.0 (Thermo Fisher) to perform peak alignment, peak picking, and quantitation for each metabolite. Next, peak intensities were normalized to the total spectral intensity and matched with the mzCloud (https://www.mzcloud.org/) and ChemSpider (http://www.chemspider.com/) databases to obtain accurate qualitative and relative quantitative results. Statistical analyses were performed using the statistical software packages R (version R-3.4.3), Python (version 2.7.6), and CentOS (release 6.6).

These metabolites were annotated using the KEGG database (http://www.genome.jp/kegg/). Principal-component analysis was performed using meta X (flexible and comprehensive software for processing metabolomics data). Metabolites with a VIP (variable importance in projection) (represents the contribution rate of differences in metabolites among different groups) score of ≥1, a *P* value of <0.05, and a fold change of ≥1.25 or ≤0.8 (14) were considered to be differentially expressed between two groups. Volcano plots were used to filter metabolites of interest, which were based on the log_2_(fold change) and −log_10_(*P*) values of the metabolites. The functions of these metabolites and metabolic pathways were studied using the KEGG database. Metabolic pathway enrichment of the differential metabolites was performed. When the ratio *x*/*n* > *y*/*N* was satisfied, the metabolic pathway was considered enriched (where *x* is the number of differential metabolites associated with this pathway, *n* is the number of differential metabolites annotated by KEGG, *y* is the number of all metabolites associated with this pathway, and *N* is the number of all metabolites in the KEGG annotation).

### Subjects.

We recruited 120 children with ASD and 60 TD children again in this study to verify the results in mice. ASD was diagnosed by a developmental pediatrician or psychologist through a series of structured interviews according to DSM-5 criteria. The exclusion criteria included a history of other developmental disorders, neurological or psychiatric diseases, genetic metabolic disease, major physical illness, recent infections, and the use of medications, antibiotics, antifungals, probiotics, or prebiotics within 3 months before the sampling. The TD children were age (2 to 6 years), gender, region, height, weight, and socioeconomic status matched with the ASD group. They also received health examinations and were proven to be free from any signs of developmental disorders or mental illness. The exclusion criteria were the same as those used for the ASD group. Participation in this research was voluntary. Parents signed written informed consent forms and were willing to let their children participate in the study. Fresh fecal samples from the children in the ASD and TD groups were collected in clean collection tubes and immediately frozen at −80°C. The methods of metabolism detection were the same as those used for the animal experiments.

### Western blot analysis.

Western blot (WB) analysis was carried out as previously reported (64). Total protein was extracted from the colon and prefrontal cortex of the mice. Approximately 40 μg protein per lane was loaded onto a 10% SDS-polyacrylamide gel (Beyotime, China) and electrophoretically separated, and the proteins were then transferred to polyvinylidene fluoride membranes (Millipore, USA). Following blocking with 5% bovine serum albumin (Gen View, USA), the membranes were incubated with primary antibodies, including TPH1 (1:1,000; Abways, China), TPH2 (1:1,000; Abways, China), 5-HT1AR (1:1,000; Abways, China), SERT (1:1,000; Abcam, CA), and actin (1:1,000; Santa Cruz, CA) antibodies, at 4°C overnight, followed by incubation with hydrogen peroxide-conjugated secondary antibodies (1:5,000; Santa Cruz, CA) at room temperature for 1 h. Next, the protein bands were detected using a chemiluminescent hydrogen peroxide substrate (Millipore, Burlington, MA). Images of the bands were captured using a Syngene GBox imaging system (Gene Company, China).

### Statistical analysis.

GraphPad Prism (version 5.0) was used to analyze the data. All data are expressed as means ± standard errors of the means (SEM). Significant differences were calculated using Student’s *t* test unless otherwise indicated. The statistical significance level was set at a *P* value of <0.05.

### Data availability.

The raw reads were deposited in the NCBI database (BioProject accession number PRJNA664898).
