# Supplementary figures and images for: Fecal Microbiome Transplantation from Children with Autism Spectrum Disorder Modulates Tryptophan and Serotonergic Synapse Metabolism and Induces Altered Behaviors in Germ-Free Mice
Source: mSystems. 2021 Apr 6;6(2):e01343-20. doi: 10.1128/mSystems.01343-20 (PMC8547010; doi:10.1128/mSystems.01343-20)

**
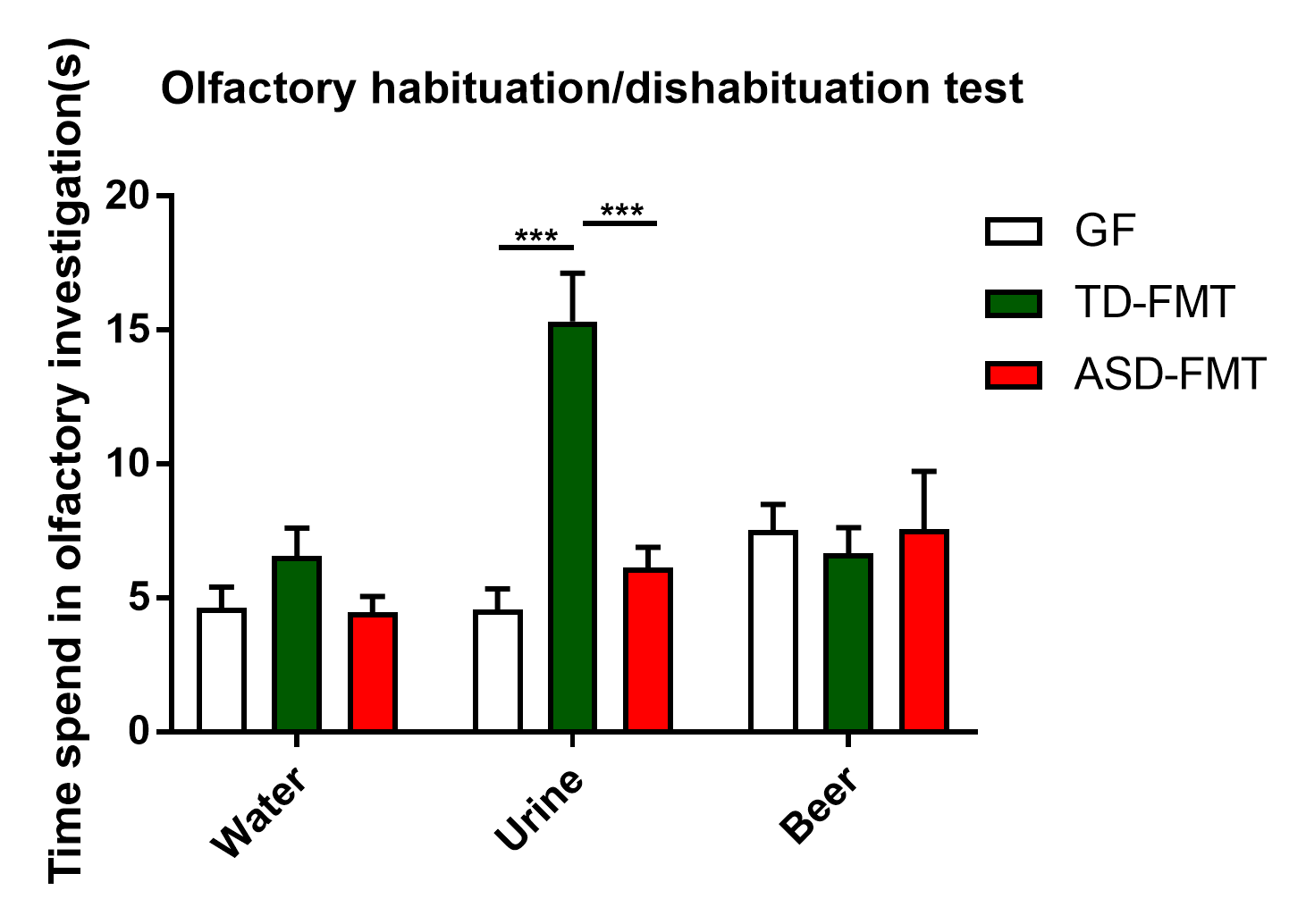
**

Supplement: FIG S1 [file msystems.01343-20_sf001.doc]
